# Protection against Bronchiolitis Obliterans Syndrome Is Associated with Allograft CCR7^+^CD45RA^−^ T Regulatory Cells

**DOI:** 10.1371/journal.pone.0011354

**Published:** 2010-06-29

**Authors:** Aric L. Gregson, Aki Hoji, Vyacheslav Palchevskiy, Scott Hu, S. Samuel Weigt, Eileen Liao, Ariss Derhovanessian, Rajeev Saggar, Sophie Song, Robert Elashoff, Otto O. Yang, John A. Belperio

**Affiliations:** 1 Division of Infectious Diseases, Department of Medicine, University of California Los Angeles, Los Angeles, California, United States of America; 2 UCLA AIDS Institute, University of California Los Angeles, Los Angeles, California, United States of America; 3 Division of Pulmonary and Critical Care, Department of Medicine, University of California Los Angeles, Los Angeles, California, United States of America; 4 Department of Biomathematics/Biostatistics, University of California Los Angeles, Los Angeles, California, United States of America; 5 Department of Pathology and Laboratory Medicine, University of California Los Angeles, Los Angeles, California, United States of America; 6 Department of Microbiology, Immunology, and Molecular Genetics, University of California Los Angeles, Los Angeles California, United States of America; University of Pittsburgh, United States of America

## Abstract

Bronchiolitis obliterans syndrome (BOS) is the major obstacle to long-term survival after lung transplantation, yet markers for early detection and intervention are currently lacking. Given the role of regulatory T cells (Treg) in modulation of immunity, we hypothesized that frequencies of Treg in bronchoalveolar lavage fluid (BALF) after lung transplantation would predict subsequent development of BOS. Seventy BALF specimens obtained from 47 lung transplant recipients were analyzed for Treg lymphocyte subsets by flow cytometry, in parallel with ELISA measurements of chemokines. Allograft biopsy tissue was stained for chemokines of interest. Treg were essentially all CD45RA^−^, and total Treg frequency did not correlate to BOS outcome. The majority of Treg were CCR4^+^ and CD103^−^ and neither of these subsets correlated to risk for BOS. In contrast, higher percentages of CCR7^+^ Treg correlated to reduced risk of BOS. Additionally, the CCR7 ligand CCL21 correlated with CCR7^+^ Treg frequency and inversely with BOS. Higher frequencies of CCR7^+^ CD3^+^CD4^+^CD25^hi^Foxp3^+^CD45RA^−^ lymphocytes in lung allografts is associated with protection against subsequent development of BOS, suggesting that this subset of putative Treg may down-modulate alloimmunity. CCL21 may be pivotal for the recruitment of this distinct subset to the lung allograft and thereby decrease the risk for chronic rejection.

## Introduction

Lung transplantation is a therapeutic option for end-stage lung disorders, but is complicated by allograft rejection with an incidence and severity that is among the highest of solid organ transplants [1]. Long-term survival is largely dependent upon recipients remaining free of bronchiolitis obliterans syndrome (BOS). BOS is a chronic alloimmune mediated, fibro-obliterative syndrome characterized by progressive airflow obstruction and graft dysfunction [2]–[5]. BOS affects over 60% of lung transplant recipients within five years after transplantation [1], [6], [7] and imparts a 3-year mortality of > 50% [1]. Over the last 20 years nearly 10,000 lung transplants have been performed in the USA, suggesting that over 6,000 individuals have developed and 3,000 died from BOS; a tremendous human and financial burden [8]. Despite BOS being a major obstacle to long-term survival post-lung transplantation, there is presently no effective means of early detection, prevention, or treatment [9].

The regulatory T lymphocyte (Treg, CD3^+^CD4^+^CD25^hi^Foxp3^+^) is recognized as a cell integral to protection against autoimmunity and allograft rejection via the down-regulation of cellular immunity [10]–[17]. Treg are believed to suppress the activity of alloreactive, effector CD4^+^ and CD8^+^ T cells, and thereby contribute to allograft survival [18]–[21]. To our knowledge, no studies have examined the frequency of bronchoalveolar lavage fluid (BALF) Treg subsets or the chemokines responsible for their recruitment and accumulation in the lung allograft.

We recently found no difference in BALF Treg (CD4^+^CD25^hi^Foxp3^+^) frequencies and ratios to effector T cells (CD8^+^CD38^+^) in lung transplant recipients with or without rejection [22], although some recipients with increased Treg during rejection did not go on to develop BOS (unpublished data). We therefore hypothesized that allograft Treg may be protective against BOS. Herein we describe our characterization of Treg subsets and chemokine protein expression in BALF from a larger cohort of lung transplant recipients with known BOS outcomes.

## Materials and Methods

### Study Design and Patient Population

Forty-seven participants underwent routine screening bronchoscopy with transbronchial biopsy and were recruited non-consecutively between December 2006 and December 2008 from patients in the UCLA Medical Center Heart and Lung Transplantation Program, who had undergone single, bilateral or combined heart and lung transplantation. For this cross-sectional analysis seventy BALF were randomly collected for Treg and Treg subset analysis at times post-transplantation that were not pre-specified. In April of 2009 the BOS status of recipients for whom BALF was analyzed by FACS was determined as described below; there was no pre-specified follow-up time.

### Ethics Statement

Each participant provided written, informed consent under a University of California, Los Angeles Institutional Review Board-approved protocol that specifically approved of this study.

### BALF Processing

Thirty to fifty mL of bronchoalveolar lavage fluid was immediately placed on ice after collection and processed within six hours. BALF was filtered through sterile 4×4 inch cotton gauze into sterile 50 mL conical centrifuge tubes and spun down, and the eluate was saved for protein analysis. The cell pellet was washed with 30 mL of sterile phosphate buffered saline solution (PBS) and then viably cryopreserved in fetal calf serum (FCS) with 10% DMSO (Sigma) for later batch analysis. Control experiments were consistent with our previous published observations and showed that fresh versus frozen cells yielded similar results [22]. The BALF fraction taken for protein analysis was re-centrifuged for 10 minutes at 500x*g*. The cell-free solution was aliquoted and frozen immediately at −70°C until thawed for chemokine ELISA.

### Cell Staining for Flow Cytometry

Frozen BALF cells were thawed in R10 (RPMI, 10% heat-inactivated FCS, HEPES, triple antibiotic) then resuspended in PBS and allowed to incubate for 20 minutes at 37°C with LIVE/DEAD Blue (Invitrogen). The reaction was quenched with the addition of R10. BALF cells were resuspended in PBS with 4% FCS and 0.1% sodium azide, and incubated with the following monocolonal antibodies for 30 minutes at 4°C: anti-CD3-AmCyan (BD Biosciences), anti-CD4-APC-H7 (BD Pharmingen), anti-CD25-AlexaFluor647 (Biolegend), anti-CD45RA-Cy5.5PerCP (eBioscience), anti-CCR7-Cy7PE (BD Pharmingen), anti-CCR4-phycoerythrin (PE) (R&D Systems) and anti-CD103-fluorescein isothiocyanate (FITC) (eBioscience). Cells were then stained with intracellular anti-Foxp3-Pacific Blue (clone PCH101) (eBioscience) as per the manufacturer's recommendations, followed by washing and fixation in 1% paraformaldehyde. Acquisition was performed using a LSR II (BD Biosciences) cytometer with the following configuration: 1) Blue laser (488 nm) with the following filters 785LP (780/60), 685LP (695/40), 600LP (610/20), 550LP (575/26), 505LP (525/50); 2) Red laser (640 nm) with 755LP (780/60), 685LP (710/50), 660/20; 3) Violet laser (405 nm) with 635LP (660/40), 595LP (605/40), 505LP (525/50), 450/50; and 4) UV light (355 nm) with 505LP (525/50) and 450/50. Compensation was performed using singly-stained tubes of each conjugated fluorochrome and BD CompBeads (BD Biosciences). Sphero Rainbow Fluorescent Particles (3.0–3.4micron) (Spherotech) were used for laser and detector calibration prior to each flow run.

### Flow Cytometry Analysis

**Figure 1:**
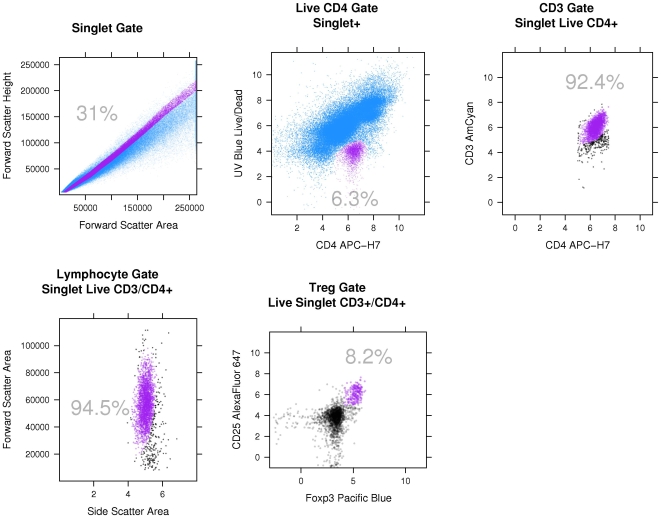
Representative gating strategy for identification of CD3^+^CD4^+^CD25^hi^Foxp3^+^ lymphocytes and subsets. Gated cells (selected cells) are purple, while non-gated cells (discarded cells) are blue or black. The gating involves sequential boolean filters and the sequence of filters is demonstrated here moving left to right and then top to bottom. Purple cell populations are selected in each scatterplot and this population is then used as the parent population in the subsequent scatterplot. (top row) First, singlets are identified in a forward scatter-area vs. forward scatter-height plot and gated on prior to logicle transformation of the data. Live/CD4^+^ cells are then identified in a CD4 vs. UV-Blue plot. CD3^+^ cells are then identified in a CD4 vs. CD3 plot. (bottom row) Lymphocytes within this population are identified in a side scatter-area vs. forward scatter-area plot. Treg are identified in a Foxp3 vs. CD25 plot. Determination of gates for CD25, CD45RA, Foxp3 and CCR7 utilized fluorescence minus-one (FMO) tubes.

**Figure 2:**
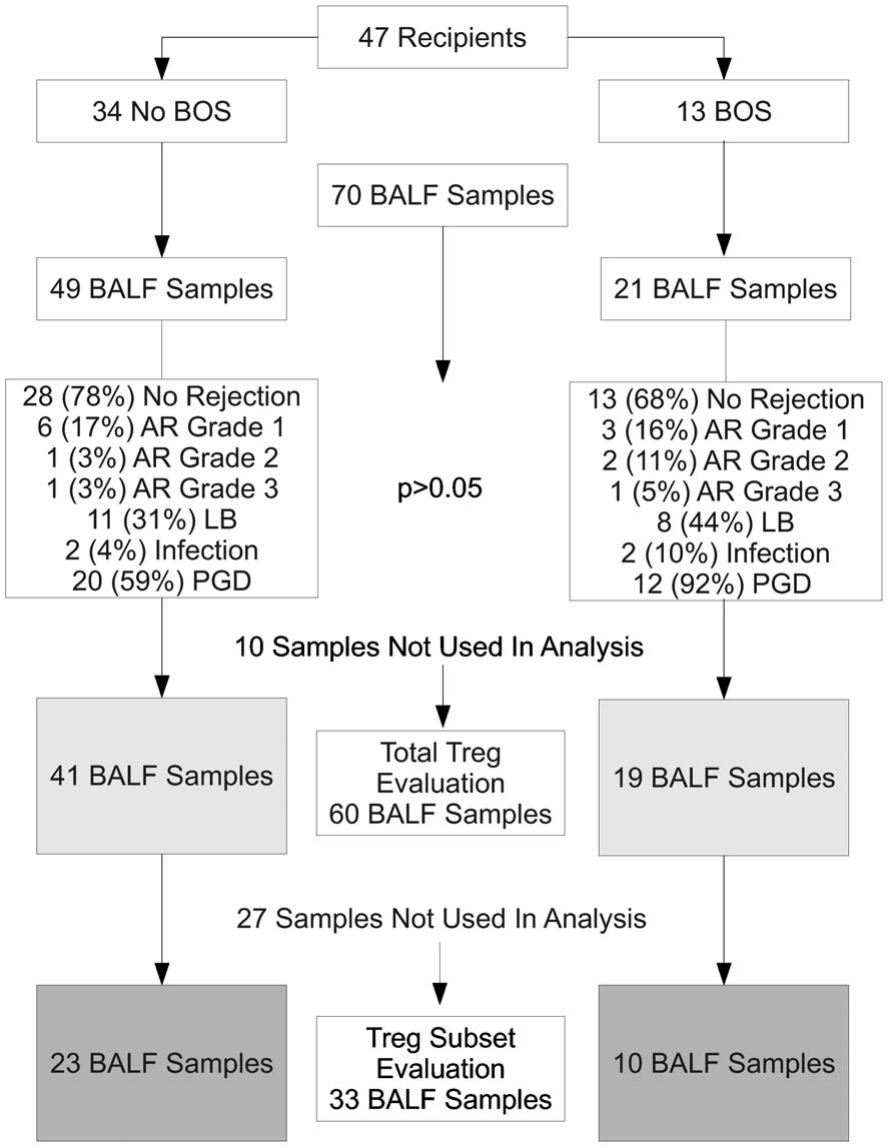
Flow chart description of the number of samples and outcomes from each group. Forty-seven lung transplant recipients were in the study. Thirteen went on to develop BOS. Seventy BALF samples were collected and ten had insufficient cells to allow detection of Treg, thus 60 samples were used for total Treg analysis. Of the 60 samples, 27 were dropped from analysis for the Treg subsets due to inadequate number of Treg as explained in the Methods.

The regulatory T lymphocyte (Treg defined as CD3^+^CD4^+^CD25^hi^Foxp3^+^ herein) and subsets expressing CCR4, CCR7, CD103 (the integrin alpha_E_beta_7_) and/or CD45RA were determined. A representative gating strategy is shown in Figure 1. Singlets were identified in a forward scatter-area (FSC-A) vs. forward scatter-height (FSC-H) plot and gated on prior to logicle transformation of the data [23]. Live/CD4^+^ cells were identified in a CD4 vs. UV-Blue plot. CD4^+^/CD3^+^ cells were then identified in a CD4 vs. CD3 plot. CD4^+^ lymphocytes within this population were identified in a side scatter-area (SSC-A) vs. FSC-A plot. Treg were identified in a Foxp3 vs. CD25 plot. Determination of gates for CD25, CD45RA, Foxp3, CCR7, CCR4 and CD103 was done by corresponding fluorescence minus-one (FMO) control tubes. In addition, an internal control of Live CD3^+^CD4^−^CD25^hi^Foxp3^+^ lymphocytes was used to determine background staining for Live CD3^+^CD4^+^CD25^hi^Foxp3^+^ lymphocytes (Treg). The lowest limit of detection above this background was pre-specified as 2*SD of the number of positive events within the Treg gate in this internal control population. These methods were required to statistically account for the rare event analysis of putative Treg subsets. Ten BALF samples of 70 were excluded from the analysis of total Treg due to poor sample quality as assessed by flow cytometry, leaving 60 samples for total Treg analysis (Figure 2). For analysis of Treg subsets, a further 27 samples were excluded due to insufficient Treg as per our pre-specified detection limit, leaving 33 samples for Treg subset analysis. There was only one BALF sample per recipient in the Treg subset analyses.

### ELISA Assays

Human CCL19, CCL21, CCL17 and CCL22 protein levels from the unconcentrated BALF samples were quantified using DuoSet ELISA development kits (R&D Systems, Minneapolis, MN). We attempted to recover BALF protein from samples after we had run FACS. Unfortunately, 23 samples went through a freeze-thaw which causes degradation of chemokine proteins. Thus nine samples from our Treg subset analysis were available for BALF chemokine protein analysis.

### Immunohistochemical Staining

For immunohistochemical staining slides were “deparafined” by incubation at 60°C for 30 minutes followed by immersion in xylene then in decreasing concentrations of ethanol (100, 95, 70%) as previously described [24], [25]. The slides were placed in a pressure cooker with citrate buffer for 30 minutes for antigen retrieval, prior to rinsing with de-ionized water. Slides are then placed in the modified endogenous blocking solution (PBS +3% H_2_0_2_) for 10 minutes. Antibody labeling was achieved by first flooding the slide with appropriate blocking solution (3% normal rabbit serum) and incubating for 30 minutes. The primary antibody was applied (anti-human CCL21 antibody (R&D Systems) or the appropriate isotype control IgG) and incubated for 30 minutes at room temperature, overnight at 4°C, then 30 minutes at room temperature, followed by a series of washes with PBS + tween. The slides were then flooded with Vectastain Elite ABC reagent (Vector Laboratory, Inc.) and allowed to incubate 40 minutes. After a PBS + tween rinse, the horseradish peroxidase-bound avidin-biotin was applied and DAB solution was added until development had reached desired level, then the reaction was quenched with water. The counterstaining was accomplished by dipping the slide in hematoxylin (Biomeda), rinsing then dipping in 10% ammonia and the rinsing in escalating concentrations of ethanol culminating in a xylene rinse.

### Acute Rejection, BOS and Immunosuppression

Acute rejection (AR) and BOS, ≥ Stage I, was determined according to standard criteria by investigators blinded to the experimental study results [26]. Patients were placed on pre- and post-transplantation immunosuppression of tacrolimus (8–20 mg/mL 12 hour trough), mycophenolate mofetil (MMF) 1000 mg orally BID (dose adjusted for toxicity), prednisone (intra-operative methylprednisolone 7 mg/kg times one, then 125 mg every 12 hours for three doses, then 0.5 mg/kg daily for one week post-transplantation with a 5 mg per week taper to a baseline of 5–15 mg daily) and prophylactic antibiotics (trimethoprim-sulfamethoxazole 160 mg BID Mondays and Thursdays and valganciclovir 450–900 mg daily). Unless contraindicated, all patients received induction therapy with either rabbit anti-thymocyte-globulin (1.5 mg/kg on post-operative days 0, 1 and 2) or, if over the age of 65 or at increased risk of malignancy or infection as perceived by the transplant physician, basiliximab (20 mg on post-operative days 0 and 4). Rapamycin was not initiated prior to the diagnosis of BOS in any study patient. Asymptomatic AR grade A1 was treated with recycling of oral prednisone at the discretion of the attending pulmonologist. Clinically significant/symptomatic AR > =  grade A1 was treated with pulse methylprednisolone, thymoglobulin, basiliximab, alemtuzumab, and/or IVIg at the physician's discretion.

### Statistical Analysis

Data collection and cell quantification was performed in a blinded fashion. The Chi-square test of association for categorical variables, the nonparametric Wilcoxon rank-sum tests for continuous variables over time and Kendall's Rank Correlation test to measure correlations between variables and ordinal outcomes have been applied. The Cox proportional hazards model was used to assess differences in time to BOS between variables of interest with interval censoring. Descriptive statistics are presented as medians [25th, 75th quantiles]. There was no adjustment for multiplicity in this exploratory analysis. The ROC curve was drawn to test the power of the predicted values from the logistic regression model, to discriminate between positive and negative cases. All tests were two sided and performed in R [27]–[29] and BioConductor [30]–[32].

## Results

### Lung Transplant Patient Population and BOS Outcomes

Seventy BALF samples were collected prospectively on 47 lung transplant recipients who underwent routine screening after transplantation (Figure 2). These samples were collected at a median of 91.5 [7, 184] days post-transplantation. Concurrent transbronchial biopsies were available for the 70 BALF collections with 14 episodes of AR, including nine grade 1, three grade 2, two grade 3, and 19 with lymphocytic bronchitis (many samples had concurrent lymphocytic bronchitis and rejection).

Recipients were followed for a median of 633 [291,732] days post-transplantation to determine BOS outcome. Thirteen recipients (28%) developed BOS at 254 [231, 457] days. The ages of the subjects with and without development of BOS were similar (59 [54, 62] vs. 59 [54, 64], p = 0.98). The timing of sample collection after transplantation was also similar between those with and without development of BOS (113 [40, 270] vs. 63 [7, 183] days, p = 0.10). Four samples had concurrent infections with two bacterial pneumonias in each group. Further descriptors for all recipients with samples included in the Treg subset (33) analysis is given in Table 1.

**Table 1:** Descriptive statistics by BOS outcome for Treg subset analysis (CCR7^+^ and CCR4^+^ subsets).

	No BOS N = 23	BOS N = 10	Test Statistic
Lymphocytic Bronchitis	13% (2)	60% (6)	X^2^ = 6, p = 0.01^1^
Rejection	22% (5)	30% (3)	X^2^ = 0.26, p = 0.61^1^
Infection	4% (1)	10% (1)	X^2^ = 0.39, p = 0.53^1^
Age	58[49, 61]	60[54, 62]	F_1,31_ = 0.17, p = 0.67^2^
Male Sex	43% (10)	50% (5)	X^2^ = 0.12, p = 0.73^1^
Pre-Tx Diagnosis:			X^2^ = 13.6, p = 0.06^1^
Cystic Fibrosis	0% (9)	10% (1)	
COPD	39% (9)	0% (0)	
IPF	39% (9)	60% (6)	
LAM	0% (0)	20% (2)	
Bronchiectasis	4% (1)	0% (0)	
Pulmonary HTN	4% (1)	0% (0)	
Sarcoid	9% (2)	0% (0)	
Scleroderma	4% (1)	10% (1)	
Acute Rejection Grade:			X^2^ = 2.29, p = 0.52^1^
0	67% (10)	70% (7)	
1	27% (4)	20% (2)	
2	7% (1)	0% (0)	
3	0% (0)	10% (1)	
Sample Day Post-Tx	61[1, 167]	97[48, 293]	F_1,31_ = 02.25, p = 0.14^2^

N is the number of non-missing values.

Numbers after percents are frequencies.

a[b, c] represents the median a, lower quartile b and the upper quartile c for continuous variables.

Tests used:

1 Pearson test;

2 Wilcoxon test.

### Total CD3^+^CD4^+^CD25^hi^Foxp3^+^ Lymphocyte (Treg) Population Did Not Predict Subsequent BOS

**Figure 3:**
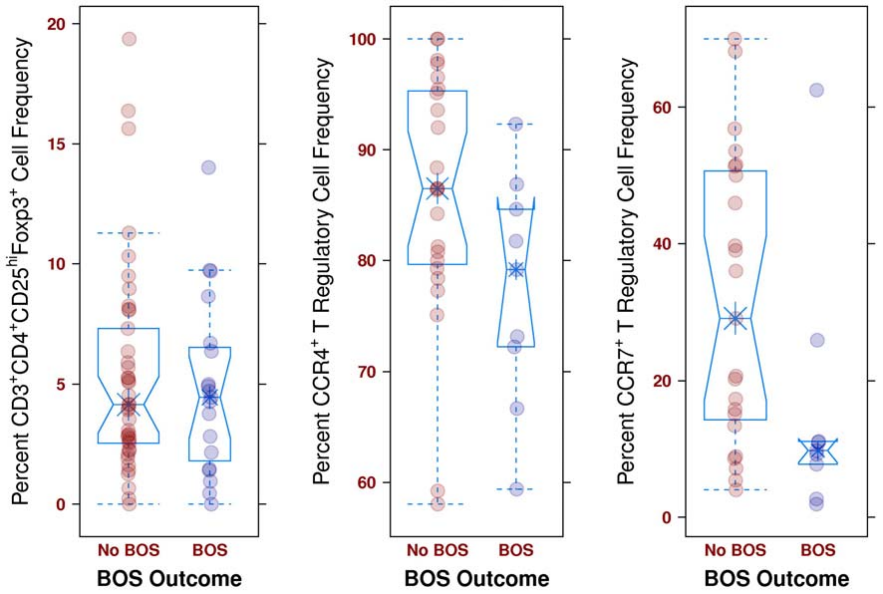
Boxplots of T regulatory lymphocytes and BOS outcomes. (left) A boxplot of all 60 evaluable samples for total Treg. This plot demonstrates that there are no differences in total Treg frequencies between those recipients who eventually develop BOS and those that do not (p = 0.92). (middle) A boxplot of all 33 evaluable samples for Treg subsets. This plot demonstrates that there are no differences in CCR4^+^ Treg frequencies between those recipients who eventually develop BOS and those that do not (p = 0.12). (right) A boxplot of all 33 evaluable samples for Treg subsets. The CCR7^+^ subset is associated with protection against BOS (p = 0.04).

**Figure 4:**
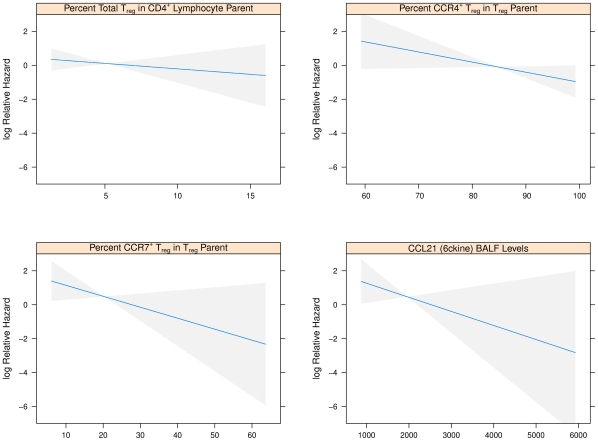
Plots of the log Cox Relative Hazard ratio for BOS against percent T regulatory lymphocyte subsets. The log of the Cox Relative Hazard (y-axis) decreases, i.e., less likely to develop BOS, as the percent of Treg, Treg subset or CCL21 protein level (x-axis) increases. The steeper the curve, as indicated by the blue line, the more protective the association with each Treg subset. The most significant effect is seen with the CCR7^+^ Treg subset. Confidence intervals are given by the gray bands. (top left) A plot for total BALF Treg, which demonstrates a non-significant protective effect. (top right) A plot of percent CCR4^+^ Treg, which demonstrates no significant protective effect. (bottom left) A plot of percent CCR7^+^ Treg, which demonstrates a significant protective effect. (botom right) A plot of BALF CCL21 protein level (pg) as determined by ELISA, which demonstrates a significant protective effect.

The frequencies of CD3^+^CD4^+^CD25^hi^Foxp3^+^ lymphocytes (Treg) in BALF from routine post-transplant bronchoscopies were assessed (Figure 2). Consistent with the reported memory phenotype of Treg, essentially all of these cells were CD45RA^−^ (100% [99.4, 100]). Comparing samples from subjects who subsequently developed BOS to those who did not during follow-up, there was no significant difference in the frequency of this total Treg population (4.5% [1.8, 6.5] vs. 4.2% [2.5, 7.3], p = 0.92) (Figure 3). This relationship was further evaluated with the Cox Proportional Hazards (Cox) model to account for the time from sample collection to the development of BOS. In the Cox model we found no association in either direction (HR = .0, p = 0.96) (Figure 4). Limiting analysis to those samples taken during documented acute rejection (14 subjects) also demonstrated no difference in Treg frequencies between recipients with or without development of BOS (p = 0.76). There was no correlation with total Treg and time of sample post-transplantation (tau = −0.002, p = 0.98).

### Subpopulations of Treg with CCR4 or CD103 Did Not Correlate With Development of BOS

**Figure 5:**
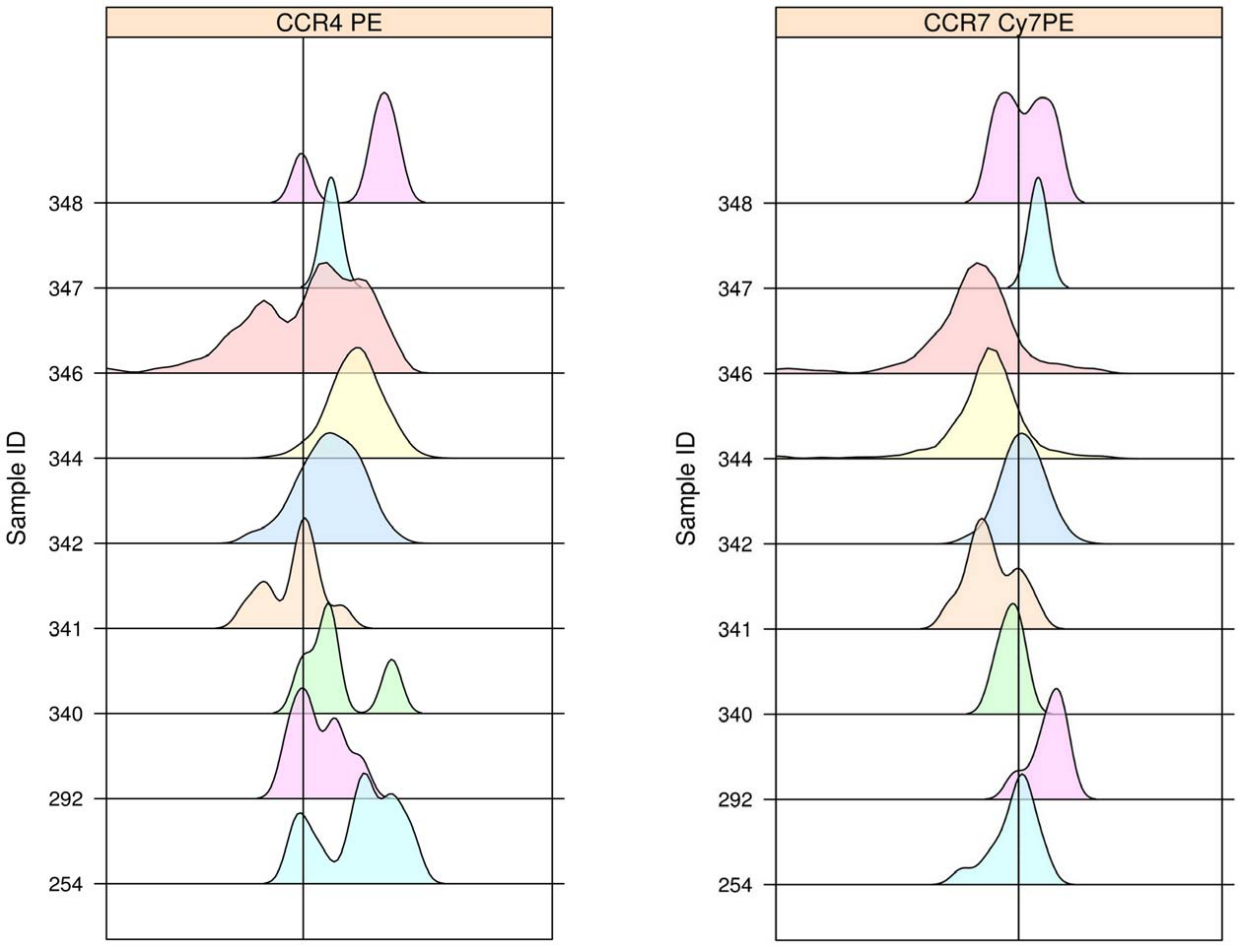
Density plots of chemokine receptor expression by Treg. Representative density plots of CCR4 and CCR7 expression by BALF Treg. The parent populations for determination of chemokine receptor positivity were singlet, live, CD3^+^CD4^+^CD25^hi^Foxp3^+^ lymphocytes as shown in Figure 1. (left) Demonstrates the generally high level of CCR4 expression by Treg. The CCR4 gate was determined such that <0.1% of the cells were positive in the CCR4 FMO tube (see text for detail). (right) Demonstrates the variability of CCR7 expression by Treg. The CCR7 gate was determined such that <0.3% of the cells were positive in the CCR7 FMO tube.

We next considered subpopulations of Treg that might be better indicators of immunoregulatory activity, with the hypothesis that recruitment and retention within the allograft is crucial for effective Treg activity against rejection. Subsets of Treg defined by CCR4 (a pivotal chemokine receptor shown to recruit Treg to inflammatory sites and allografts) [33], [34] or CD103 (an alpha_E_beta_7_ integrin involved in trafficking of T cells to inflammatory sites) [35], [36] were assessed for prediction of BOS. The majority of Treg expressed CCR4 (Figure 5), but Treg expression of CCR4 did not demonstrate a significant difference between recipients with and without development of BOS during study follow-up (77.5% [70.9,85.2] vs. 86.5% [79.6,95.4], p = 0.12) (Figure 3). We further found no association of CCR4 expression on Treg and the subsequent development of BOS in the Cox model (HR = 0.94, p = 0.08) (Figure 4). There was no trend of CCR4 expression with time of sample after transplantation (tau = −0.04, p = 0.73). Virtually none of the CD3^+^CD4^+^CD25^hi^Foxp3^+^CD45RA^−^ lymphocytes expressed CD103 (0.0% [0.0, 0.0]). Thus, neither CCR4 nor CD103 expression was reflective of Treg subsets that potentially protected against BOS.

### CCR7 Expression on Treg is Associated With Protection From BOS

As studies have noted that CCR7 can be expressed by Treg and may be critical for Treg trafficking between lymphatic and allograft tissue [34], we determined if CCR7 was expressed on our BALF Treg. Overall, the expression of CCR7 on Treg was variable between subjects, with a median of 18.8% [9.1, 47.0] (Figure 5). Notably, the median frequency of the CCR7^+^ Treg subset was significantly higher in those that did not develop BOS than in those that did subsequently develop BOS (29.1% [14.3, 50.7] vs. 9.8% [7.8, 11.1] p = 0.04) (Figure 3). Importantly, this finding was also supported by the Cox model which accounted for sample collection time in relation to the development of BOS (HR = 0.94, p = 0.04) (Figure 4). The expression of CCR4 by CCR7^+^ versus CCR7^−^ Treg was similar (89.2% [69.1, 100] vs. 84.2% [72.9, 95.7], p > 0.10), indicating that CCR4 did not play a major role in distinguishing trafficking of these subsets to the lung allograft. There was not a significant trend of CCR7 expression with time of sample collection post-transplantation (tau = −0.02, p = 0.88). These results suggest that CCR7 is a marker of a key subset of Treg that may protect against BOS.

### CCL21 Protein Levels in BALF Correlate with CCR7^+^ Treg Frequencies

Having observed that higher frequencies of CCR7^+^ Treg in lung allograft BALF are associated with reduced incidence of BOS, we sought to identify potential chemokines that drive recruitment and retention of these cells in the allograft. The two known ligands for CCR7, CCL21 (6ckine) and CCL19 (MIP-3beta), were assessed by ELISA. BALF CCL21 protein levels correlated with frequencies of CCR7^+^ Treg (tau = 0.34 and p = 0.03), while CCL19 did not (tau = −0.11, p = 0.47). Given the putative importance of CCR4 for Treg recruitment, we also assessed the CCR4 ligands CCL17 (TARC) and CCL22 (MDC). There was no correlation to CCR4^+^ or CCR7^+^ subsets nor total Treg frequencies. We further assessed for a correlation between CCL21 protein levels and BOS outcome and found an inverse association (tau = −0.64 and p = 0.04) (see Figure 4). These results underscore the likely importance of Treg recruitment to the lung allograft via chemotaxis mediated by the interactions of CCL21 and CCR7.

**Figure 6:**
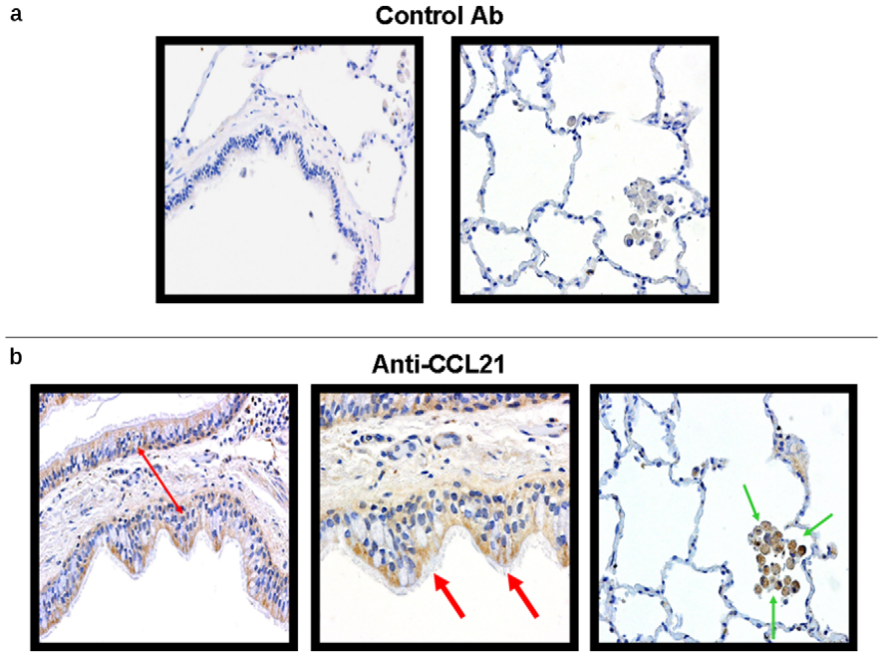
Histopathology demonstrating CCL21 production in healthy lung allograft. Representative immunohistochemistry for (a) control antibody and (b) CCR7 ligand CCL21 on healthy lung allograft biopsy tissue. CCL21 protein expression is found in bronchial epithelial cells (red arrows) and alveolar macrophages (green arrows). Panels were photographed at 200× and 400× magnification.

Our finding of an association between CCL21 and CCR7^+^ Treg in BALF led us to investigate the cellular sources of CCL21 in healthy lung allografts. Multiple biopsies (n = 5) from healthy lung allografts without infection or rejection demonstrated that CCL21 protein is predominately expressed from bronchiolar epithelial cells and alveolar macrophages (Figure 6).

## Discussion

**Figure 7:**
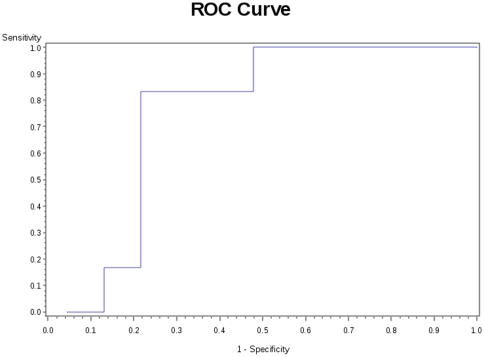
ROC curve for CCR7^+^ Treg and BOS. The ROC curve is based upon a logistic regression model for CCR7+ Treg predicting BOS outcome, which excludes samples collected after the diagnosis of BOS. The p-value for the global null hypothesis is 0.04 and the area under the curve (AUC) is 0.75; the model has good predictive value.

In this study we describe a unique subpopulation of CD3^+^CD4^+^CD25^hi^Foxp3^+^CD45RA^−^ (Treg) that express CCR7 and are associated with protection against the future development of BOS after lung transplantation. This finding offers a potential cellular biomarker that could be useful in early prediction of BOS and provides potential insight into the mechanism of chronic rejection (Figure 7). Such a biomarker would be a means with which to limit over-immunosuppression and its associated morbid risks of infections and cancer.

Our findings suggest that CCR7^+^ Treg are important for protection against BOS. This is in agreement with a recent murine model of islet cell transplantation, wherein maximum suppression of allograft rejection required the expression of CCR7 on Treg [34]. Most of our Treg, including the CCR7^+^ Treg, are CCR4^+^, consistent with murine transplantation models that demonstrate CCR4 expression on Treg in the allograft [33], [34]. However, we find no differences in terms of BOS outcome with the CCR4^+^ subpopulation. In fact, neither of the CCR4 ligands correlate with Treg or subpopulation frequencies, suggesting that CCR4 may not be critical for the maintenance of important Treg subsets within the allograft. We do demonstrate that the CCR7 ligand CCL21 is expressed by lung allograft mononuclear and bronchial epithelial cells and levels in BALF correlate with frequencies of the CCR7^+^ Treg subset. Additionally, BALF CCL21 appeared protective against the development of BOS. This data suggests that the interaction between CCL21 and CCR7^+^ Treg is a pivotal biologic axis for Treg recruitment to the lung allograft and for allowance of protection against BOS.

CD103 is the alpha_E_beta_7_ integrin which binds e-cadherin and is reportedly present on intraepithelial lymphocytes [35], [36]. Previous investigators had noted low overall expression of CD103 by human, peripheral CD4^+^CD25^+^ regulatory lymphocytes [37]–[40] consistent with our findings herein, suggesting that CD103 may be an important marker for murine Treg but not for human Treg [41]–[44].

It should be mentioned that our study has several caveats. This is not a longitudinal study and BALF samples were not collected at a uniform time post-transplantation, which may have introduced unknown biases. While we are able to identify Treg within the BALF of >87% of the samples, most samples had fewer than 20 Treg (hundreds of lymphocytes), which led to a significant decline in evaluable samples from 70 to 60 for total Treg evaluation and from 60 to 33 for Treg subset evaluations (Figure 2). This was based on our methodology of using internal controls to set a minimum number of Treg in which subsets could be confidently assessed. All samples were collected prospectively and longitudinally, but only a few of the 47 subjects have more than one sample in this cohort preventing identification of within patient trends over time. Importantly, we did not find any trend in Treg or Treg subset frequencies based on the time of sample collection post-transplantation. Due to the small numbers of Treg recovered from the samples we are not able to sort and perform functional assays to measure suppressive capacity of these CD3^+^CD4^+^CD25^hi^Foxp3^+^CD45RA^–^CCR7^+^ cells. This is an inherent weakness in the study of human BALF samples, which we hope to overcome in future studies. Furthermore, by the time we analyzed our BALF chemokine protein levels all but nine samples had undergone a freeze thaw process. Thus our analysis for correlation between BALF protein levels and Treg subsets and BOS should be interpreted with caution.

To our knowledge, this study is the first to utilize multiparameter flow cytometric analysis of BALF lymphocytes in lung transplant recipients. Multiparameter FACS is necessary to exclude non-Treg by using live-dead discrimination dye and FMO tubes. An internal control is also used to define the appropriate detection limit in the rare subset event analysis. While it remains possible that the cells we have captured within the BALF are distinct from the parenchymal lung cells, prior publications have noted that such cells are nearly identical both functionally and phenotypically [45].

The importance of an accurate marker to identify lung transplant recipients not at risk for BOS cannot be over-emphasized. Rejection is pervasive post-lung transplantation and even one episode of A1 has been suggested to increase the subsequent risk of BOS [46]–[48], as have primary graft dysfunction and HLA mismatch [46], [49]–[51]. Augmentation of immunosuppression is the usual response to rejection, but it increases the risk of infection, which may increase long-term risk for BOS [46], [52]. Yet to be described are protective factors within the lung allograft that could help to direct potentially harmful adjunctive immunosuppressive therapy away from lung transplant recipients with reduced risk of BOS.

In conclusion, we describe a novel CCR7^+^ Treg subpopulation that may be recruited to the lung allograft by CCL21 and which correlates with the delayed onset of or protection against development of BOS. While preliminary, these data suggest that a CCR7^+^ subset of Treg is involved in the prevention of chronic graft dysfunction after lung transplantation. Further studies are underway to validate this finding in a larger, longitudinal cohort of patients and to assess the functionality of these cells.
